# A Radiosensitivity Gene Signature and XPO1 Predict Clinical Outcomes for Glioma Patients

**DOI:** 10.3389/fonc.2020.00871

**Published:** 2020-06-16

**Authors:** Shan Wu, Qiao Qiao, Guang Li

**Affiliations:** Department of Radiation Oncology, The First Hospital of China Medical University, Shenyang, China

**Keywords:** glioma, XPO1, nomogram, prognosis, radiosensitivity

## Abstract

**Objective:** Glioma is the most common and fatal primary brain tumor that has a high risk of recurrence in adults. Identification of predictive biomarkers is necessary to optimize therapeutic strategies. This study investigated the predictive efficacy of a previously identified radiosensitivity signature as well as Exportin 1 (XPO1) expression levels.

**Methods:** A total of 1,552 patients diagnosed with glioma were analyzed using the Chinese Glioma Genome Atlas and The Cancer Genome Atlas databases. The radiosensitive and radioresistant groups were identified based on a radiosensitivity signature. Patients were also stratified into XPO1-high and XPO1-low groups based on *XPO1* mRNA expression levels. Overall survival rates were compared across patient groups. Differential gene expression was detected and analyzed through pathway enrichment and Gene Set Enrichment Analysis (GSEA). To predict 1-, 3-, and 5-years survival rates for glioma patients, a nomogram was established combining the radiosensitivity gene signature, XPO1 status, and clinical characteristics. An artificial intelligence clustering system and a survival prediction system of glioma were developed to predict individual risk.

**Results:** This proposed classification based on a radiosensitivity gene signature and XPO1 expression levels provides an independent prognostic factor for glioma. The RR-XPO1-high group shows a poor prognosis and may benefit most from radiotherapy-combined anti-XPO1 treatment. The nomogram based on the radiosensitivity gene signature, XPO1 expression, and clinical characteristics performs more optimally compared to the WHO classification and IDH status in predicting survival rates for glioma patients. The online clustering and prediction systems make it accessible to predict risk and optimize treatment for a special patient. The cell cycle, p53, and focal adhesion pathways are associated with more invasive glioma cases.

**Conclusion:** Combining the radiosensitivity signature and XPO1 expression is a favorable approach to predict outcomes as well as determine optimal therapeutic strategies for glioma patients.

## Introduction

Glioma is one of the most common malignant primary brain tumors in adults that is known for a high recurrence risk and fatality. After surgery and adjunct therapies such as radiotherapy and chemotherapy, the survival rates for patients diagnosed with glioma vary widely (1). This variation has been considered to result from histological grade. However, with progress in genetic and molecular biology, molecular biomarkers were discovered and the WHO classified gliomas based on histological and molecular parameters (2). Due to the high heterogeneity and intricacy, a single biomarker does not fully characterize tumor properties for gliomas and more accurate predictions using multi-parameter markers is required. Thus, a multivariate tool is needed to predict the prognosis and to guide proper treatment for glioma.

A “31-gene signature” identified from four different published microarrays in NCI-60 cancer cell lines represents radiosensitivity (3). This signature has been validated in many malignancies including GBM and LGG cohorts (3–7). However, the predictive significance of the 31-gene signature in glioma patients is unclear and is not a signature currently applied in clinical practice. Exportin 1 (XPO1) is a special specific carrier protein that transports tumor suppressor proteins such as p53, p73, FOXO, pRB, BRCA1, and PP2A. Expression levels of XPO1 are elevated in cancer cells, which leads to excessive nuclear export and dysfunction of tumor suppressor proteins. XPO1 overexpression also correlates with poor prognosis and radiotherapy resistance in some cancers (8–12). Therefore, XPO1 has been identified as an oncogenic target and an inhibitor against XPO1 has been approved for the treatment of multiple myeloma. For glioma patients, XPO1 is a promising treatment modality and is being tested in clinical trials (NCT02323880, NCT01986348). However, further understanding is required to develop effective clinical XPO1-targeted therapeutic strategies against glioma.

This study aimed to establish a promising predictive tool for glioma prognosis and to distinguish specific patients who can benefit from combinational radiotherapy and XPO1 inhibition.

## Materials and Methods

### CGGA and TCGA

Clinicopathological data and transcriptome sequencing data for glioma samples were obtained from three publicly available data sets including the Chinese Glioma Genome Atlas (CGGA http://www.cgga.org.cn/), The Cancer Genome Atlas (TCGA https://xenabrowser.net/), and GSE16011 data sets (GEO https://www.ncbi.nlm.nih.gov/geo/query/acc.cgi?acc=GSE16011).

Transcriptome sequencing data on CGGA for glioma samples were generated using the Illumina Hiseq platform. After excluding patients that did not have survival data (*n* = 81), radiotherapy (*n* = 87), or histological type (*n* = 5) data recorded as well as those that did not contain gene expression information (*n* = 1), a total of 889 cases were included in the CGGA cohort. For TCGA data, the gene expression profile was measured using the Illumina HiSeq platform. After excluding cases that did not contain survival data (*n* = 12) or intact gene expression information (*n* = 6), a total of 663 cases were included in the TCGA cohort. A total of 1,552 glioma samples informative for overall survival were included in this analysis. After excluding patients without follow-up information, a total of 264 cases were included in GEO cohort.

### Group Clustering

Based on the expression profile for the 31-gene signature (31-GS), the CGGA and TCGA cohorts were divided into two groups using the k-means method. Clusters with poor progress were defined as the radioresistant group (RR groups) and the others were considered as radiosensitive groups (RS groups).

*XPO1* mRNA expression levels were analyzed to determine XPO1 expression. The package “survivalROC” was used to divide samples into XPO1 high-expression vs. XPO1 low-expression group (13). Differences between XPO1-high and XPO1-low groups were depicted using bar graphs. Clinicopathological features between the two groups were compared. Furthermore, samples were defined into additional cohorts including the RR-XPO1-high, RR-XPO1-low, RS-XPO1-high, and RS-XPO1-low groups. Overall survival (OS) was analyzed for all groups. Multivariate analysis was used to identify independent factors associated with prognosis.

### Nomogram Building and Validating

The CGGA cohort was defined as the primary cohort and the TCGA cohort and GEO cohort were identified as the validation cohorts. A nomogram was established based on results from the multivariate analysis conducted by the “rms” package in R version 3.6. External validation was used to confirm predictive accuracy of OS in the validation cohort. The C-index was applied to assess the accuracy of three predictive systems including the nomogram (CNS WHO classification) and CNS WHO classification combined with the mutation status of isocitrate dehydrogenase (IDH). Differences between the predicted probabilities of the nomogram and actual outcomes were evaluated using the calibration curve. To translate our research into clinical application, we constructed an artificial intelligence clustering system and a survival prediction system of glioma. These tools are available online.

### Discovery of Associated Biological Pathways

To explore the underlying mechanisms linked to XPO1 expression and radiosensitivity, differentially expressed genes (DEGs) between the RR-XPO1-high and RS-XPO1-low groups were separately identified using the “edgeR” R package. When there was a false discovery rate (FDR) *q* < 0.01 and fold change (FC) > 2.0, significant genes were defined (Supplementary Images 1A,B). Similarities of significant genes in both the CGGA and TCGA cohorts were classified as significant DEGs (Supplementary Images 1C,D). Next, upregulated and downregulated PPI networks were built using Cytoscape software (version 3.6.0). The Cytoscape plug-in Molecular Complex Detection (MCODE) was applied to investigate modules in protein–protein interaction (PPI) networks. Another Cytoscape plug-in termed ClueGo was used to detect corresponding Kyoto Encyclopedia of Genes and Genomes (KEGG) pathways where genes in notable modules were enriched. Gene Set Enrichment Analysis (GSEA) was used to validate KEGG pathways uncovered using GSEA software (version 4.0.1).

### Statistical Analyses

Statistics were performed using the SPSS (version 23.0) and R Studio software (version 1.1.453). The χ^2^ test or Fisher's exact test was used to compare categorical variables. Continuous variables were compared using the *t* test when normally distributed and the Mann–Whitney *U* test was used to compare continuous variables with an abnormal distribution. Survival curves were generated using the Kaplan–Meier method and analyzed using the log-rank test. Cox regression analysis was used for multivariate analyses.

## Results

### Validation That the 31-Gene Signature Is Related to Radiosensitivity in Glioma

**Figure 1:**
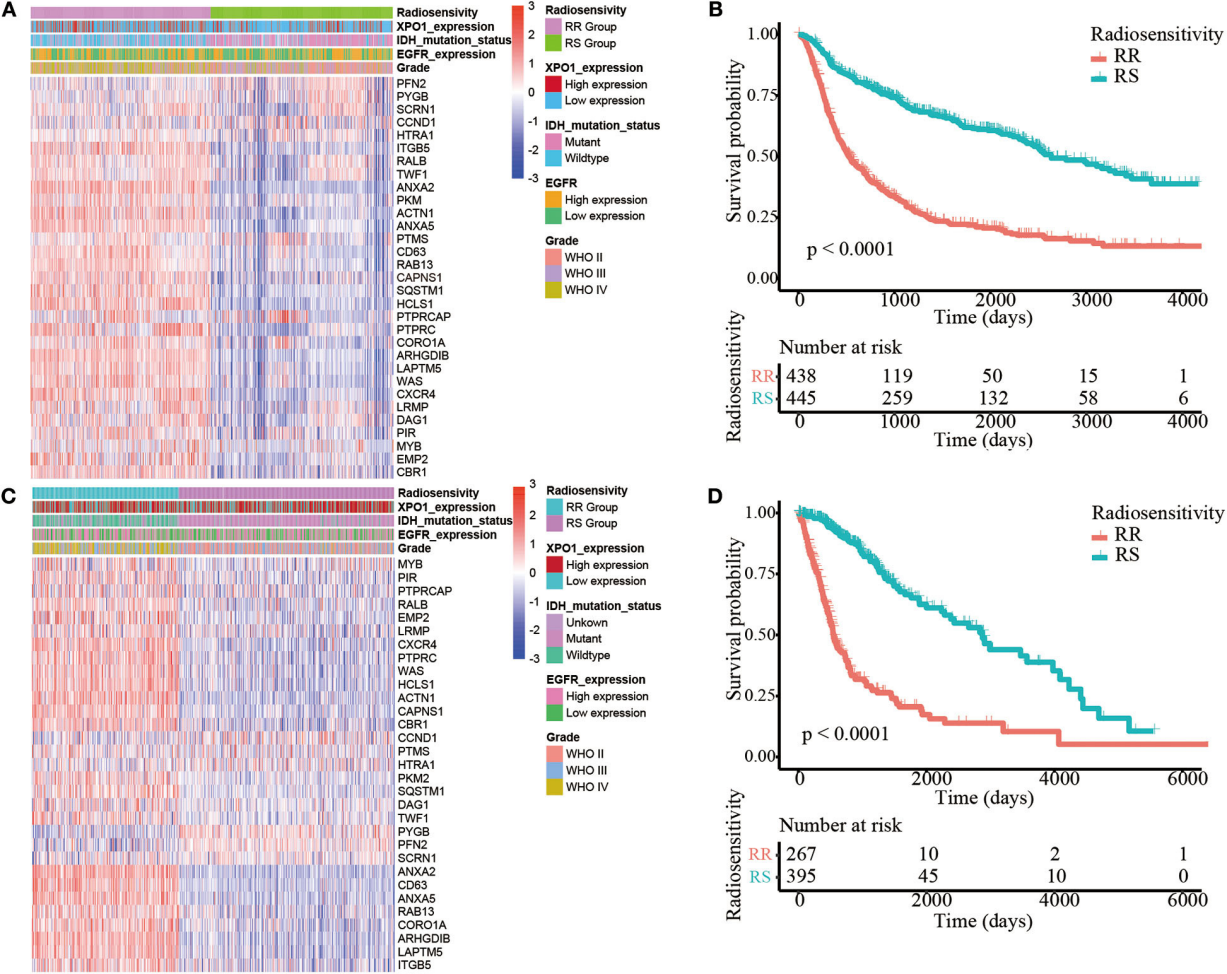
Identification of the radiosensitive (RS) and radioresistant (RR) groups. **(A)** A heatmap illustrating the 31-gene signature, XPO1 expression, ERGF expression, IDH mutation status, and WHO classification in every sample from the CGGA cohort. Columns represent samples and rows represent gene expression and clinical characters. **(B)** Differences in OS between the two clusters in the CGGA cohort. The cluster with more satisfactory OS was defined as the RS group (*P* < 0.0001). **(C)** A heatmap illustrating the 31-gene signature, XPO1 expression, ERGF expression, IDH mutation status, and WHO classification in every sample from the TCGA cohort. **(D)** Differences in OS between the two clusters in the TCGA cohort. The cluster with more satisfactory OS was defined as the RS group (*P* < 0.0001).

The 31-gene signature was identified using an integrative meta-analysis of published microarray data for NCI-60 cancer cells (3). Based on the gene signature, all patients (*N* = 889) in the CGGA cohort were divided into two clusters including cluster 1 (*N* = 442, 49.7%) and cluster 2 (*N* = 447, 50.3%) (Figure 1A). Patients in cluster 2 showed prolonged OS compared to patients in cluster 1 (HR = 3.13, 95% CI: 2.60–3.76; *P* < 0.0001). Therefore, cluster 1 in the CGGA cohort was defined as the RR group and cluster 2 was defined as the RS group (Figure 1B). However, in the TCGA cohort (*N* = 662), 267 (40.3%) cases were divided into cluster 1 and 395 (59.7%) cases were divided into cluster 2 (Figure 1C). Similarly, patients in cluster 2 exhibited prolonged OS (HR = 4.92, 95% CI: 3.76–6.44; *P* < 0.0001). Thus, cluster 1 was nominated as the RR group and cluster 2 was nominated as the RS group (Figure 1D). Clustering for GBM was shown in Supplementary Image 3.

### XPO1 Expression Levels and Clinicopathological Features

**Figure 2:**
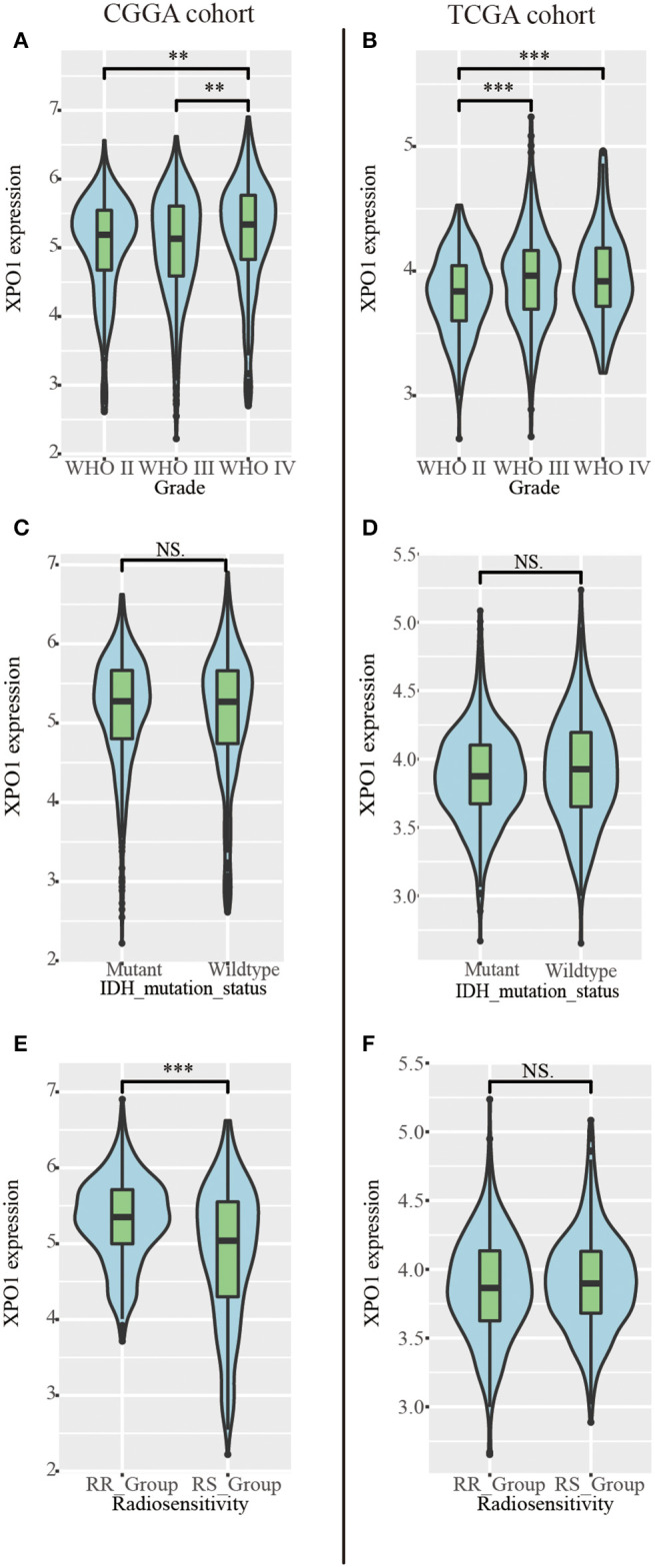
Relationship between XPO1 expression and clinicopathological features. Association between XPO1 expression and different subgroups stratified by WHO classification **(A,B)**, IDH mutant status **(C,D)**, and radiosensitivity status **(E,F)** in the CGGA and TCGA cohorts (*P < 0.05, **P < 0.01, ****P < 0.0001, ^NS^*P* ≥ 0.05).

To characterize the expression patterns of XPO1 in gliomas, RNA-sequencing data from samples in the CGGA and TCGA cohorts were analyzed. Glioblastoma (WHO IV) exhibited the highest XPO1 expression levels compared to WHO II and WHO III gliomas in the CGGA cohort (Figure 2A). Similarly, glioblastoma (WHO IV) revealed a higher expression of XPO1 compared to WHO II gliomas, with no differences compared to WHO III gliomas in the TCGA cohort (Figure 2B). These results indicated a significant positive correlation between XPO1 expression and glioma malignancy. Notably, the RR group showed significantly higher expression of XPO1 than the RS group in the CGGA (Figure 2E). However, no significant differences were found between the RR and RS groups in the TCGA cohort (Figure 2F).

**Figure 3:**
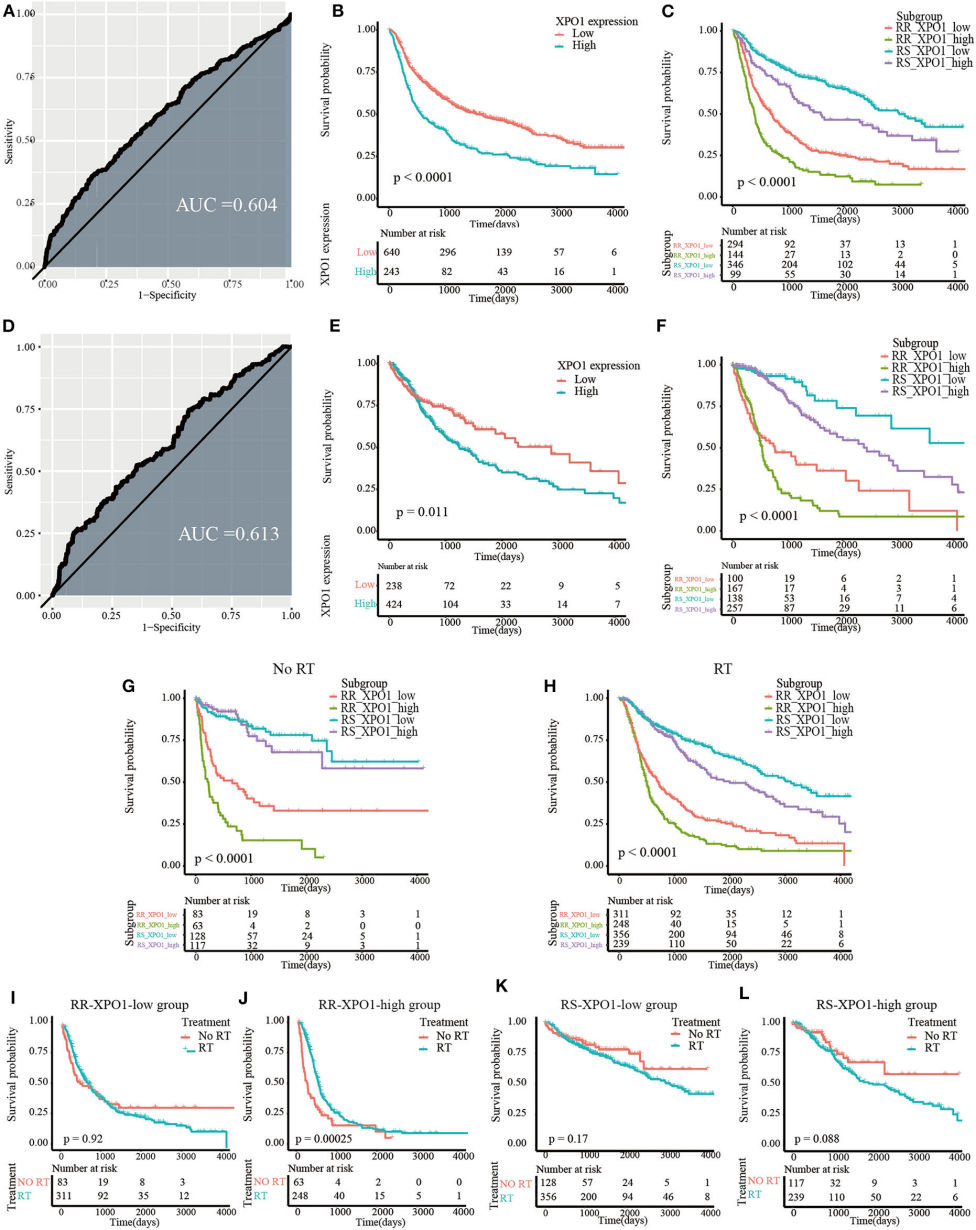
Prognostic significance of XPO1 expression and 31-GS in different subgroups. **(A,D)** Definition of XPO1-high and XPO1-low groups by the ROC curve in the CGGA and TCGA cohorts. **(B,E)** Prognostic significance of XPO1 expression in the CGGA and TCGA cohorts. **(C,F)** Prognostic differences of subgroups stratified by combining XPO1 expression and 31-GS in the CGGA and TCGA cohorts. **(G,H)** Prognostic significance of subgroups stratified by combining XPO1 expression and 31-GS for patients with and without RT. **(I–L)** Comparison of OS rates stratified by RT in the four subgroups stratified by combining XPO1 expression and 31-GS.

To explore the prognostic significance of XPO1 and define the cutoff point, a time-dependent ROC curve was used to predict 3-years OS. The AUC of the ROC curve was 0.604 for 3-years survival (Figure 3A), indicating the predictive accuracy of this prognostic model in the CGGA cohort. The AUC of the ROC curve for the TCGA cohort was 0.613 (Figure 3D). Based on the cutoff point, samples were divided into XPO1-low expression and XPO1-high expression groups. Next, the correlation between XPO1 expression levels and clinicopathological features was interrogated. As shown in Tables 1, 2, XPO1 levels did not correlate with sex, age, and IDH mutation status, but a correlation was confirmed between XPO1 levels and histological and radiosensitivity types in the CGGA cohort. Tumors with high XPO1 expression levels were enriched in higher WHO grade tumors and the RR group. The results of the TCGA cohort showed similar trends with XPO1 expression levels where there were no correlations between expression levels and sex, age, and IDH mutant status. Likewise, a correlation existed between XPO1 expression levels and histological grade. Interestingly, XPO1 expression did not correlate with radiosensitivity type in the CGGA cohort.

**Table 1:** Demographics and clinicopathologic characteristics for glioma patients in the CGGA cohort.

	**Total**	**XPO1 low**	**XPO1 high**	***P*-value**
		**expression**	**expression**	
**Sex**				
Male	530 (60%)	387 (73%)	143 (27%)	0.58
Female	359 (40%)	256 (71%)	103 (29%)	
**Age**				
<40	345 (39%)	237 (69%)	108 (31%)	0.065
≥40	543 (61%)	405 (75%)	138 (25%)	
Missing	1 (0%)	1 (100%)	0 (0%)	
**IDH_mutant_status**				
Wild type	385 (43%)	269 (70%)	116 (30%)	0.59
Mutant	460 (52%)	330 (72%)	130 (28%)	
Missing	44 (5%)	44 (100%)	0 (0%)	
**WHO_grade**				
WHO II	261 (29%)	209 (80%)	52 (20%)	0.0001
WHO III	294 (33%)	218 (74%)	76 (26%)	
WHO IV	334 (38%)	216 (65%)	118 (35%)	
**Radiosensitivity**				
RR_group	442 (50%)	295 (67%)	147 (33%)	0.0002
RS_group	447 (50%)	348 (78%)	99 (22%)	
**Radio_status**				
Yes	741 (83%)	531 (72%)	210 (28%)	0.37
No	148 (17%)	112 (76%)	36 (24%)	
**Chemo_status**				
Yes	608 (68%)	435 (72%)	173 (28%)	0.87
No	259 (29%)	187 (72%)	72 (28%)	
Missing	22 (2%)	21 (95%)	1 (5%)	

**Table 2:** Demographics and clinicopathologic characteristics for glioma patients in the TCGA cohort.

	**Total**	**XPO1 low**	**XPO1 high**	***P*-value**
		**expression**	**expression**	
**Sex**				
Male	379 (57%)	137 (36%)	242 (64%)	0.87
Female	282 (43%)	100 (35%)	182 (65%)	
Missing	1 (0%)	1 (100%)	0 (0%)	
**Age**				
<40	252(38%)	85 (34%)	167 (66%)	0.36
≥40	410 (62%)	153 (37%)	257 (63%)	
**IDH_mutant_status**				
Wild type	228(34%)	77 (34%)	151 (66%)	0.44
Mutant	419 (63%)	155 (37%)	264 (63%)	
Missing	15 (2%)	6 (40%)	9 (60%)	
**WHO_grade**				
WHO II	248 (37%)	107 (43%)	141 (57%)	0.008
WHO III	261 (39%)	87 (33%)	174 (67%)	
WHO IV	153 (23%)	44 (29%)	109 (71%)	
**Radiosensitivity**				
RR_group	267 (40%)	100 (37%)	167 (63%)	0.51
RS_group	395 (60%)	138 (35%)	257 (65%)	
**Radio_status**				
Yes	418 (63%)	138 (33%)	280 (67%)	0.044
No	244 (37%)	100 (41%)	144 (59%)	
**CHEMO_status**				
Yes	383 (58%)	122 (32%)	261 (68%)	0.011
No	279 (42%)	116 (42%)	163 (58%)	

### Both XPO1 Expression and the 31-Gene Signature Predict Overall Survival

As revealed by the Kaplan–Meier method, overexpression of XPO1 predicted a poor prognosis in the CGGA and TCGA cohorts (median survival: 18.9 months vs. 52.7 months, *P* < 0.0001 in the CGGA cohort; 40.7 vs. 94.3 months, *P* = 0.01 in the TCGA cohort) (Figures 3B,E). Furthermore, the Kaplan–Meier curve for overall survival is shown in Figures 3C,F, where the four subgroups are divided based on a combination of XPO1 expression levels (XPO1-high and XPO1-low) and radiosensitivity types (RR and RS). Differences in overall survival rates were shown for the four subgroups. (*P* < 0.0001) The RR-XPO1-high group exhibited the worst overall survival, with a median survival time of 12.7 months (95% CI: 10.2–15.4) in the CGGA cohort and 17.5 months (95% CI: 15.9–19.4) in the TCGA cohort. The best overall survival was shown for the RS-XPO1-low group, with a median survival time of 98.5 months (95% CI: 81.9–NA) in the CGGA cohort and 144.7 months (95% CI: 94.3–NA) in the TCGA cohort. The median survival of the RR-XPO1-low and RS-XPO1-high groups was 22.8 months (95% CI: 18.0–27.5) and 52.1 months (95% CI: 36.4–106.2), respectively, in the CGGA cohort and 25.1 months (95% CI: 14.7–75.0) and 79.8 months (95% CI: 57.8–113.8), respectively, in the TCGA cohort. Multivariate analysis demonstrated that age of diagnosis, IDH mutation status, histological grade, chemotherapy treatment, and radiosensitivity treatment based on signature-combined XPO1 expression status were independent risk factors for OS. Univariate and multivariate analyses are listed in Table 3.

**Table 3:** Univariate analysis and multivariate analysis of patients.

	**Univariate analysis**			**Multivariate analysis**		
	**HR**	**95% CI**	***P*-value**	**HR**	**95% CI**	***P*-value**
**Sex**						
Female	Reference					
Male	1.057	0.914–1.224	0.453			
**Age**						
<40	Reference			Reference		
≥40	2.080	1.773–2.439	<2e−16	1.237	1.039–1.474	0.017
**IDH_mutant_status**						
Mutant	Reference			Reference		
Wild type	4.524	3.879–5.275	<2e−16	1.836	1.498–2.251	4.88e−09
**WHO_grade**						
WHO II	Reference			Reference		
WHO III	2.805	2.240–3.512	<2e−16	3.055	2.380–3.921	<2e−16
WHO IV	10.147	8.152–12.629	<2e−16	6.279	4.759–8.286	<2e−16
**Radio_status**						
Yes	Reference					
No	1.189	0.9858–1.435	0.0702			
**CHEMO_status**						
Yes	Reference					
No	1.524	1.290–1.801	7.12e−07	0.691	0.576–0.830	7.36e−05
**Radiosensitivity and XPO1 status**						
RR_XPO1_high	Reference			Reference		
RR_XPO1_low	0.6473	0.5412–0.7742	1.91e−06	0.790	0.658–0.948	0.011
RS_XPO1_high	0.2483	0.1514–0.2311	<2e−16	0.583	0.451–0.754	3.75e−05
RS_XPO1_low	0.1871	0.1514–0.2311	<2e−16	0.433	0.340–0.552	1.39e−11

To investigate the efficacy of the predictive assay combining 31-GS and XPO1 expression, patients were divided into the RT and no-RT groups and survival differences were analyzed between the four subgroups defined by 31-GS and XPO1 expression. In the RT group, significant survival differences were shown for the four subgroups in pairwise comparison (*P* < 0.05) (Figure 3G). However, in the no-RT group, there were no obvious differences between survival conditions of the RS-XPO1-high and RS-XPO1-low groups (*P* = 0.60), while there were significant survival differences shown for other pairwise comparisons (Figure 3H). Moreover, the beneficial value of radiotherapy for patients in different subgroups was further investigated (Figures 3I–L). As indicated by the data, only patients in the RR-XPO1-high group were able to benefit from radiotherapy (*P* = 0.00025).

### A Nomogram to Predict OS in Glioma Patients

**Figure 4:**
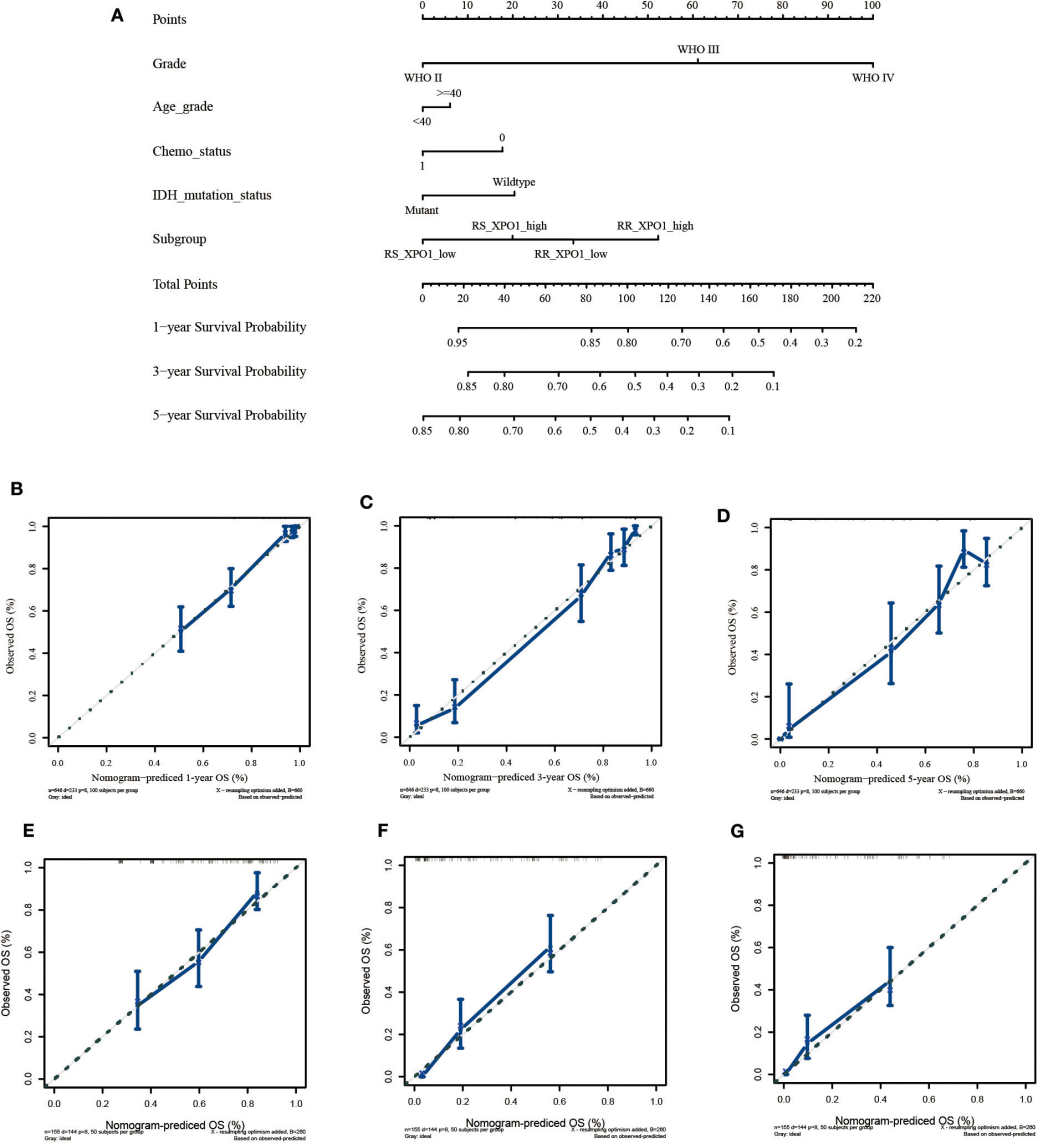
Construction and validation of the nomogram based on clinical parameters and subgroups stratified by XPO1 expression and 31-GS. **(A)** Nomogram based on clinical parameters and subgroups stratified by XPO1 expression and 31-GS. **(B–D)** Calibration curves for predicting patient survival at 1, 3, and 5 years in the TCGA validation cohort. **(E–G)** Calibration curves for predicting patient survival at 1, 3, and 5 years in the GEO validation cohort.

Since the radiosensitivity signature and XPO1 expression proved to be optimal in predicting the prognosis for glioma patients, a nomogram combining the radiosensitivity signature, XPO1 expression, and clinical characteristics was next generated. The CGGA cohort was defined as the training cohort and the TCGA cohort was defined as the validation cohort. Multivariable Cox regression analysis was used to determine independent factors for OS. According to the data, histological grade, age, chemotherapy, IDH status, radiosensitivity signature, and XPO1 expression were indicated to be predictors. A clinical survival prediction model was constructed based on the data in the training cohort and presented in a nomogram for the prediction of 1-, 3-, and 5-years survival rates (Figure 4A). Subsequently, the nomogram was externally validated using the validation set through comparison of the C-index of OS and calibration plots. In the TCGA cohort, the independent C-index of histological grade and the histological grade combined with IDH status were 0.78 (95% CI: 0.76–0.81) and 0.83 (95% CI: 0.81–0.86), respectively, which was sharply lower than the C-index of the nomogram, which was 0.86 (95% CI: 0.84–0.88) (*P* < 2.2e−16). The results for the GEO cohort were consistent with those of the TCGA cohort. The independent C-index of histological grade and the histological grade combined with IDH status were 0.64 (95% CI: 0.61–0.67) and 0.66 (95% CI: 0.62–0.69), respectively, which was sharply lower than the C-index of the nomogram, which was 0.71 (95% CI: 0.67–0.75) (*P* < 2.2e−16). As indicated by Figures 4B–G, the calibration curves for the 1-, 3-, 5-years OS rates were well-predicted in the validation cohorts.

To translate our research into clinical application, we constructed an artificial intelligence clustering system and a survival prediction system of glioma. They are available at https://online-survival-stratification-system-for-gliomas.shinyapps.io/cluster_shiny/ and https://online-survival-stratification-system-for-gliomas.shinyapps.io/dynnomapp/.

### XPO1 Expression and Associated Biological Processes

To explore the biological mechanisms associated with XPO1 overexpression in gliomas, DEGs were identified by comparing the mRNA expression profiles between the RR_XPO1_high and RS_XPO1_low groups. A total of 1222 DEGs in the CGGA and 614 DEGs in the TCGA cohort were identified, where 579 DEGs overlapped. There were 290 DEGs that were upregulated and 277 genes that were downregulated. Based on the SRTING database, PPI networks were built for upregulated genes and downregulated genes.

**Figure 5:**
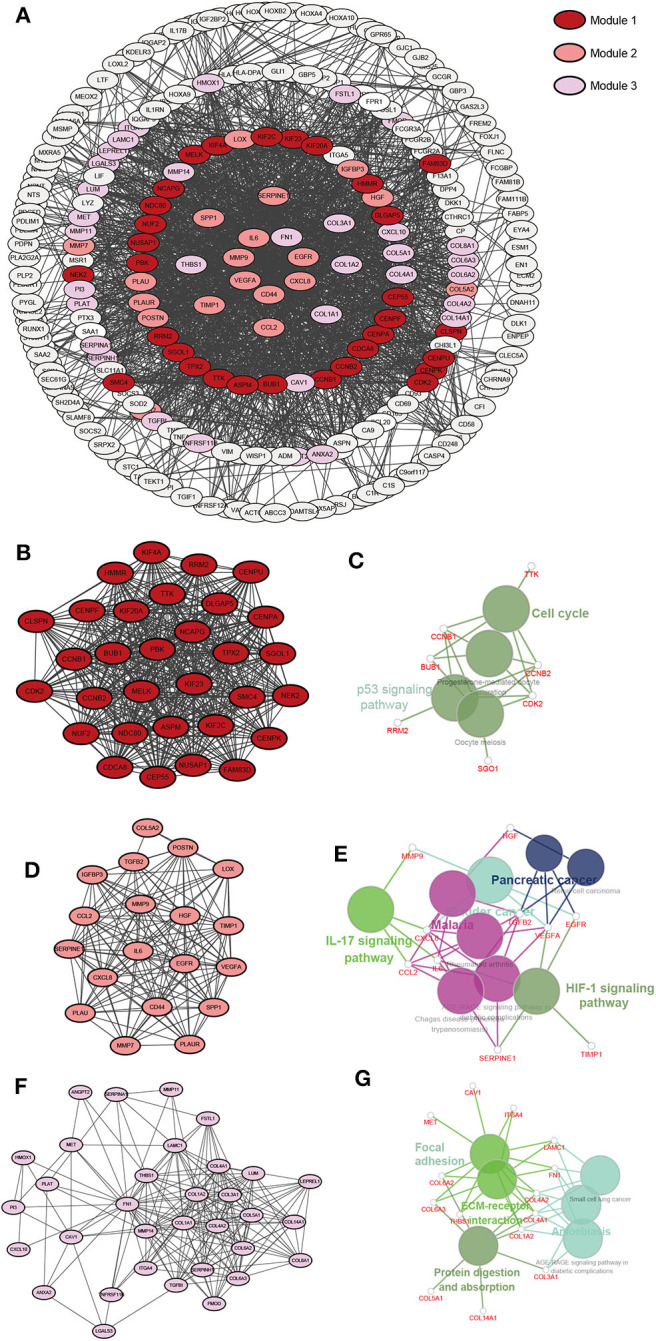
A PPI network of DEGs upregulated in the RR-XPO1-high vs. RS-XPO1-low groups. Three significant modules were identified with an MCODE score of >10. **(A)** A PPI network of GEDs that are up-regulated. **(B)** Module 1, MCODE score = 29.667. **(D)** Module 2, MCODE score = 15.556. **(F)** Module 3, MCODE score = 12.312. **(C,E,G)** Notable pathways where DEGs are enriched in module 1, module 2, and module 3.

The upregulated gene network consists of a total of 250 nodes and 2005 protein pairs (Figures 5A,C). Three modules with scores >10 were detected by MCODE. Furthermore, enrichment pathways and hub nodes of module 1, module 2, and module 3 are shown in Figures 5B–G. In module 1 (score: 29.667), the cell cycle and p53 signaling pathway were identified as significant (Figure 5C). In module 2 (score: 15.556), the IL-17 and HIF-1 signaling pathways were identified as significant (Figure 5E). In module 3 (score: 12.312), focal adhesion and ECM–receptor interaction pathways showed significant biological effects (Figure 5G).

As for the downregulated gene network, a total of 232 nodes and 1645 protein pairs were included (Supplementary Image 2A). Two modules with scores >10 were detected by MCODE (Supplementary Images 2B,D). Interestingly, there were two pathways in module 1 (score: 21.071) (Supplementary Image 2C), including pathways of nicotine addiction and synaptic vesicle cycle, that showed overlap with pathways in module 2 (score: 10.167) (Supplementary Image 2E). Genes in module 1 were also enriched in the GABAergic synapse pathway.

**Figure 6:**
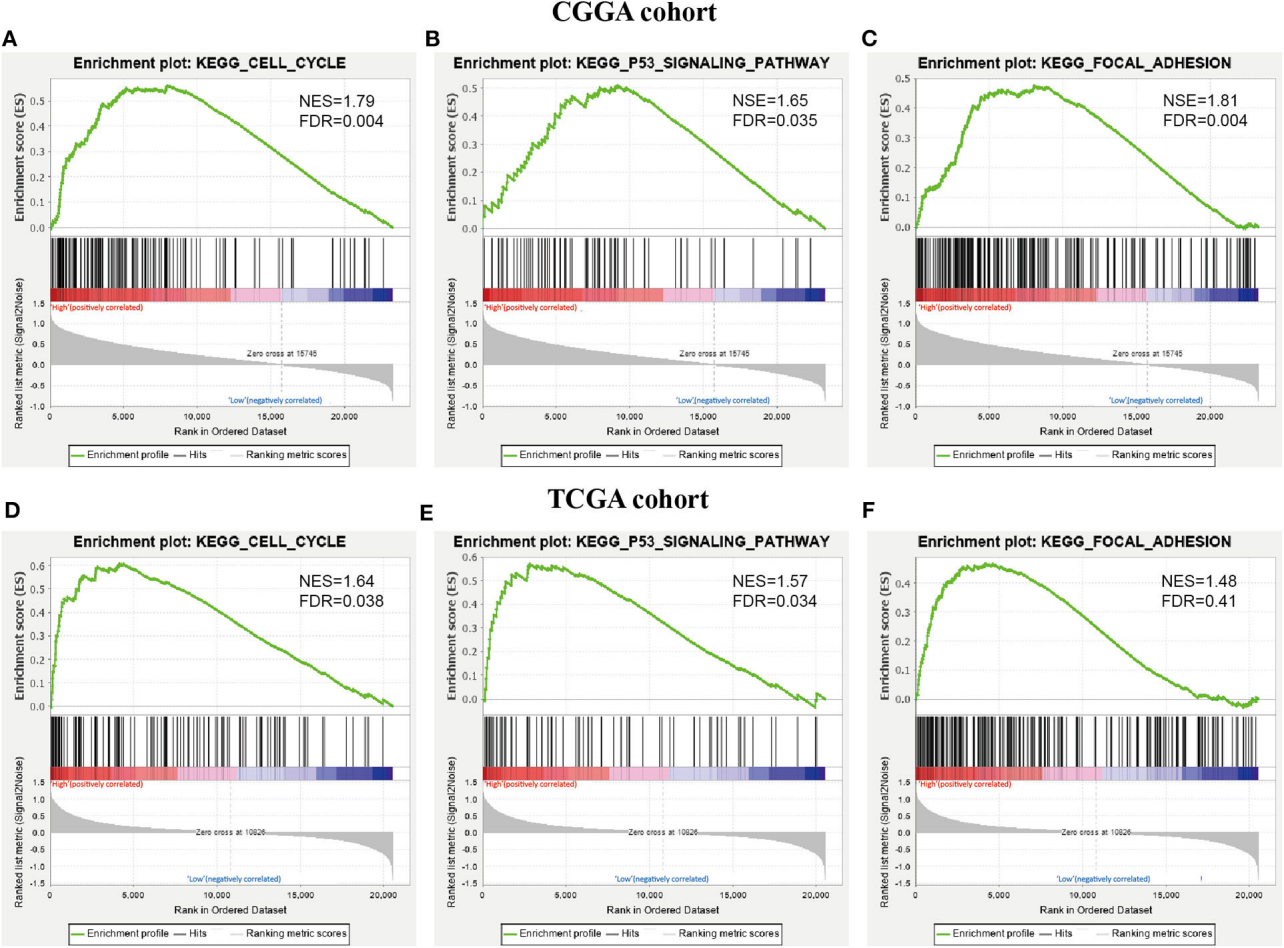
KEGG pathways confirmed by GSEA analysis. **(A–C)** GSEA was performed to confirm KEGG pathways (cell cycle pathway, p53 signaling pathway, and focal adhesion) in the CGGA cohort. **(D–F)** GSEA analysis for the three pathways in the TCGA cohort.

To further investigate the KEGG pathway and its functional roles, GSEA analysis was used to detect pathways that exhibited significant differences between RR XPO1-high and RS XPO1-low groups. As shown in Figure 6, the cell cycle, p53 signaling, and focal adhesion pathways were confirmed to be the significantly affected pathways.

## Discussion

This study established a prognostic predictor combining a 31-gene signature and XPO1 expression, which stratified glioma cases into four subgroups that were demonstrated with distinct clinical outcomes. Furthermore, a nomogram was built and proved more accurate and credible than conditional histological class and even superior than the histological class combined with IDH status when predicting the prognosis of glioma patients. Based on the previous prognostic nomogram, the study developed an artificial intelligence clustering system and a survival predictive system for individual risk prediction. These two systems were available and helpful to provide precise individual survival prediction and improve individual treatment decision-making. To our knowledge, it is the first study that detects prognostic value of 31-GS and XPO1 expression and provides a classifying strategy to determine optimal combinational treatments.

The Cancer Genome Atlas Research Network has concluded that genetic status better correlates to disease prediction in lower grade gliomas compared to histological class (14). Incorporation of molecular parameters in pathological diagnosis have been suggested by the 2016 CNS WHO classification (2). Classification systems based on histological similarities are known to induce high interobserver variation (15). In the histopathological classification of diffuse glioma, unexpectable errors may be improved due to incomplete sampling during surgery. These confusions have brought difficulties for predictive outcomes and processing of clinical decisions for glioma patients. Moreover, the 2016 CNS WHO classification is mainly based on one or two molecular parameters. Since gliomas are highly heterogeneous, the incorporation of multiple molecular signatures may be more accurate than a single biomarker to present the features of unique glioma types.

This study identified four non-overlapping molecular subtypes with significant survival differences. Stratification based on the 31-GS and XPO1 expression status proved to be an independent predictive factor for glioma. The radiosensitivity-XPO1 expression status and clinical factors were combined to establish a nomogram, which could transform statistical equations into simplified graphs, providing a reliable and convenient method for risk quantification. The predication based on our nomogram is more robust than histologic class and even superior to histological class combined with IDH status. In addition, it was also suggested by the data that more potential molecular signatures need to be uncovered and incorporated into the predictive model.

Several prognostic models have been built for predicting the survival for glioma. However, most of them display prognostic prediction for a subgroup, but fail to provide individual risk prediction for a special patient. This study built available online predictive systems, which were of clinical practical values for optimizing individual medical decision-making.

The treatment of gliomas is highly individualized and tests are available to guide the use of chemotherapy. For example, O(6)-methylguanine-DNA methyltransferase (MGMT) testing assesses drug resistance in temozolomide (TMZ)-based chemotherapy (16, 17). However, there is a lack of a diagnostic biomarker guiding adjuvant radiotherapy, in which radiosensitivity is a possible factor. The 31-GS was established to find patients who benefit from radiotherapy and has been validated to be a predictor for OS only in GBM patients with RT. Nevertheless, for prediction of radiosensitivity in LGG patients, the 31-GS requires combination with other biomarkers (4, 6). Our research, with the largest sample size of 1,552 patients, confirmed that 31-GS is an independent prognostic predictor for all glioma patients whether they received RT or not. However, the results changed after including XPO1 expression status. In the RS group, low XPO1 expression predicts prolonged OS in patients with RT. However, the prediction does not remain significant in the non-radiotherapy-treated group, which indicated that XPO1 is a predictive radiosensitivity factor only for the RS group. The XPO1-related radioresistant effect has been reported by several studies and attributed to the aberrant subcellular localization of critical proteins in apoptosis and DNA DSB repair induced by XPO1. An XPO1 inhibitor has proven to radiosensitize rectal cancer cells and GBM cells both *in vitro* and *in vivo* (12, 18). Surprisingly, only the RR-XPO1-high group was validated to benefit from RT, which conflicts with the previous hypothesis excluding the RR group from RT benefiting patients. A radiosensitivity predictor is aimed to stratify patients into three groups including those that benefit from RT, those with least aggressive tumors and favorable prognosis even without RT, and those with the most aggressive tumors and discouraging overall prognosis, which requires more intense of treatment. In our study, patients in the RR-XPO1-high group belong to the latter category. Obviously, 31-GS is not the only indicator leading to the decision of RT treatment, but after integrating XPO1, patients at high risk and who require a combinatory treatment can be stratified.

Recently, Selinexor, an oral XPO1 inhibitor, has emerged as a promising reagent for relapsed or refractory multiple myeloma (https://www.accessdata.fda.gov/scripts/cder/daf/index.cfm?event=overview.process&varApplNo=212306). As for the treatment of glioma, preclinical trials have verified that Selinexor possesses favorable CNS penetration abilities and significantly prolongs animal survival by suppressing tumor growth (19). Moreover, ongoing clinical trials are showing promising clinical value for Selinexor. One trial is a phase I study analyzing the side effects and the optimal dose for Selinexor in the treatment of younger patients with recurrent solid tumors or high-grade gliomas (ClinicalTrials.gov identifier: NCT02323880). Another trial is an open-label and multicenter Phase II trial evaluating treatment efficacy and safety of Selinexor in patients with recurrent gliomas (ClinicalTrials.gov identifier: NCT01986348). This trial, widely known as the KING study, has presented results at the 2019 ASCO annual meeting, showing that 19% of patients achieved 6 months of PFS with manageable side effects. However, the overall response rate of Selinexor in this study was only 10% and further molecular analyses need to be performed to identify enriched biomarkers. Selinexor not only suppresses glioma growth but also acts as a radiosensitizer, enhancing the radiosensitivity of rectal cancer and GBM (12, 18). Nevertheless, no clinical study has been performed combining radiotherapy and XPO1 inhibitors. The current study provides an understanding of the potential clinical value of radiosensitivity and XPO1 expression and investigates biological mechanisms behind this that can be considered for clinical trials.

In our study, poor outcomes have been confirmed in patients in the RR-XPO1-high group compared to the RS-XPO1-low group. To gain insight into intrinsic signaling pathways, DEGs between the two groups were analyzed. As a result, up-regulated DEGs in the subgroup with poor outcomes are enriched in the cell cycle, p53, and focal adhesion pathways, which was confirmed by both KEGG functional enrichment analysis and GSEA analysis. The p53 signaling and cell cycle pathways have been identified as glioma core signaling pathways (20, 21). Interestingly, p53 is a cargo protein for XPO1. Recently, a range of studies showed that inhibition of XPO1 renders p53 accumulation in the nucleus and induces apoptosis and cell cycle arrest in gliomas (11, 22–24). In addition, focal adhesion is crucial for the control of glioma cell morphology and invasion (25). Moreover, focal adhesion has also been demonstrated to function in radioresistance (26).

Despite these findings, certain limitations for this study exist. First, samples were downloaded from TCGA, CGGA, and GEO databases and information about the extent of tumor section was not provided. Since the extent of tumor section is a critical survival factor, further analysis with more detailed clinical information should be performed in future studies. Additionally, there is a possibility of selection bias as patients that did not contain all relevant information were excluded from this study.

## Conclusion

This study developed an accurate nomogram based on the combination of radiosensitivity and XPO1 expression for prognosis prediction in glioma patients. Compared to WHO stages, the nomogram exhibits superiority in accuracy and capacity in identifying individual survival predictions. Two accessible online tools, an artificial intelligence clustering system and a survival predictive system based on the novel nomogram, have been built to predict individual survival and optimize treatment decision. Combining radiosensitivity and XPO1 expression is a promising method to discover patients that may benefit from combinational therapy. As a potential strategy, the Selinexor-combined radiotherapy for gliomas is worth studying in prospective clinical trials.

## Data Availability Statement

Publicly available datasets were analyzed in this study. This data can be found here: http://www.cgga.org.cn/, https://xenabrowser.net/.

## Author Contributions

SW, QQ, and GL contributed conception and design of the study. SW performed the statistical analysis. All authors contributed to manuscript revision, read, and approved the submitted version.

## Conflict of Interest

The authors declare that the research was conducted in the absence of any commercial or financial relationships that could be construed as a potential conflict of interest.
