# Supplementary figures and images for: A Radiosensitivity Gene Signature and XPO1 Predict Clinical Outcomes for Glioma Patients
Source: Front Oncol. 2020 Jun 16;10:871. doi: 10.3389/fonc.2020.00871 (PMC7308508; doi:10.3389/fonc.2020.00871)

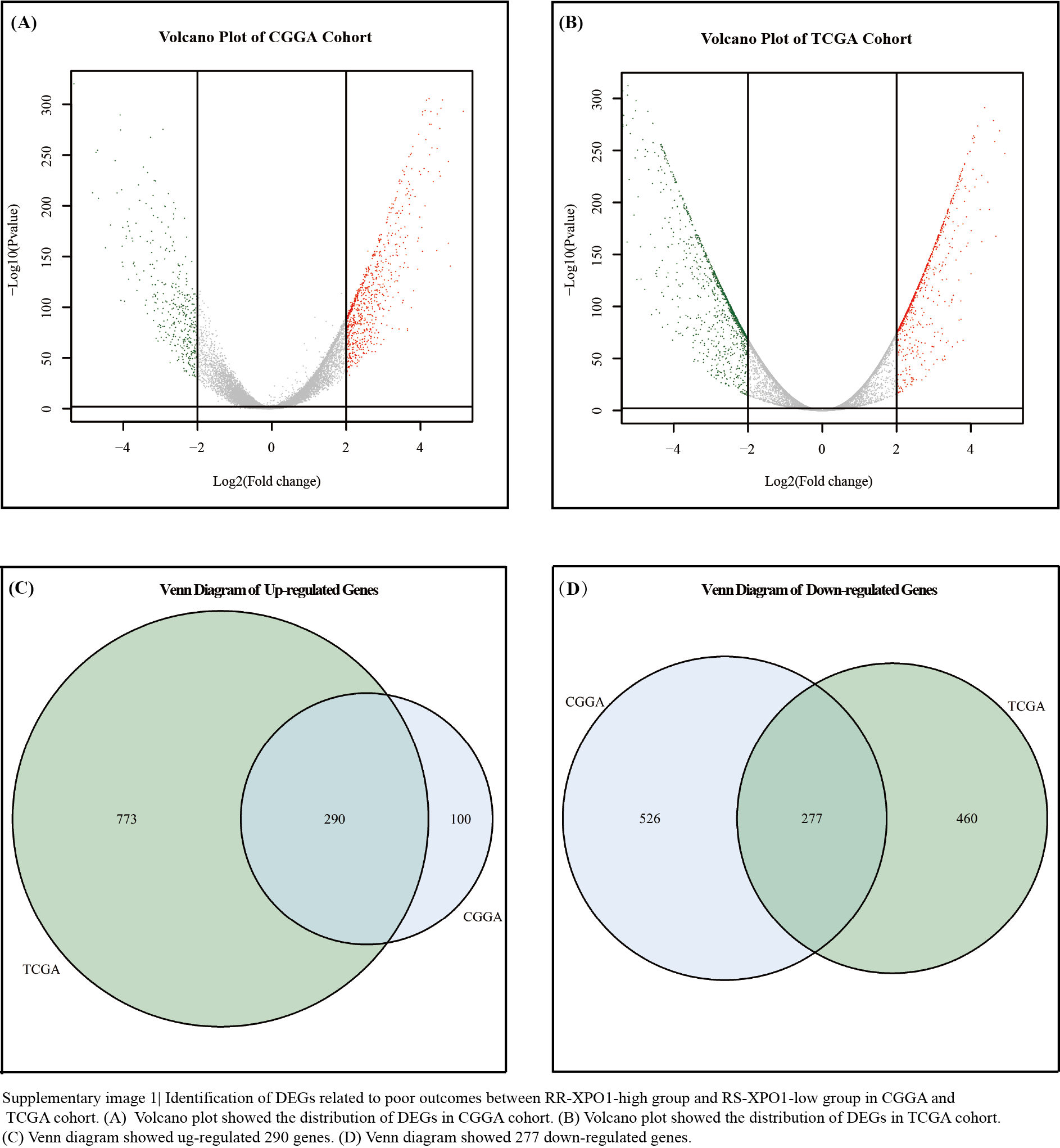

Supplement: Supplementary file 1 [file Image_1.TIF]

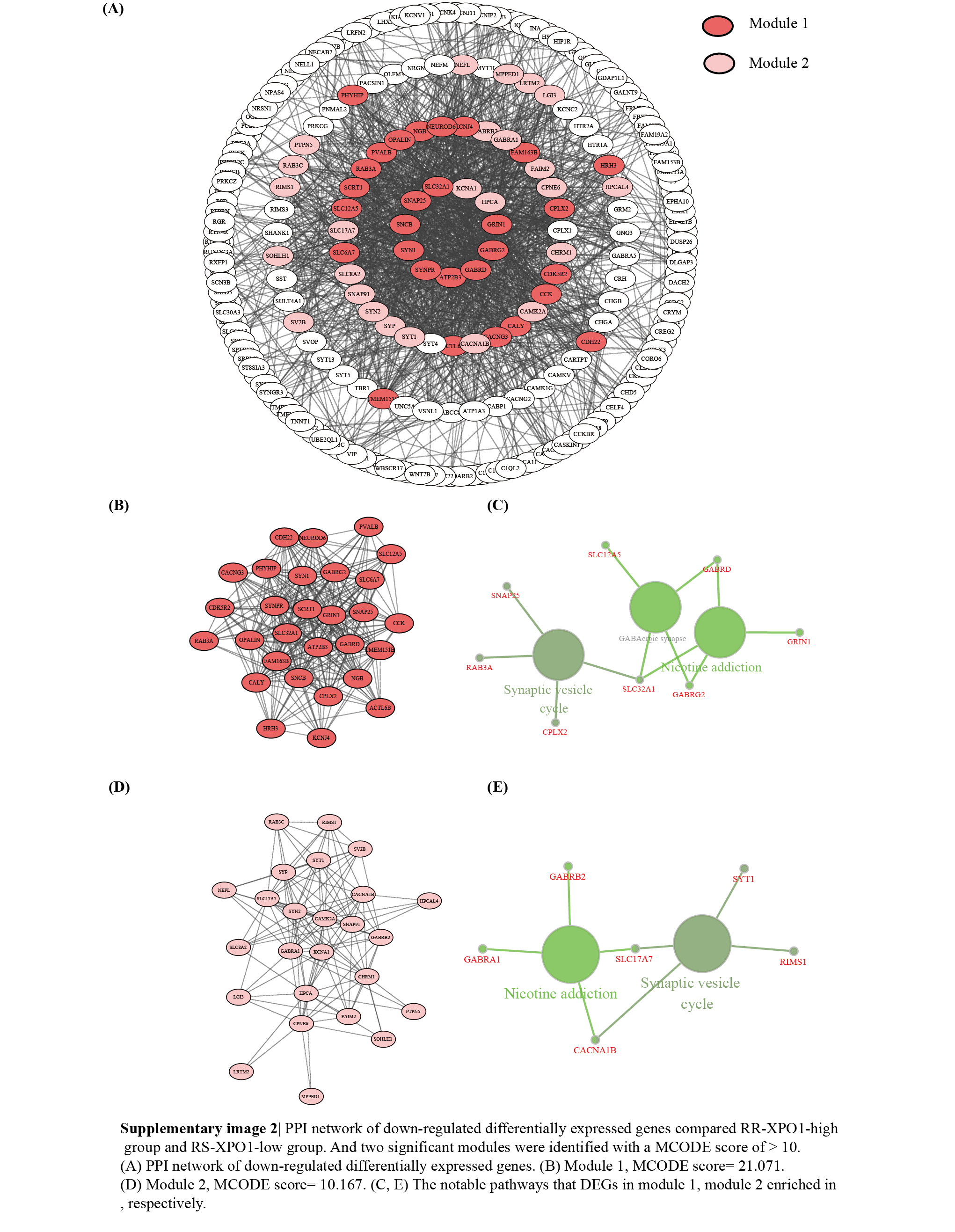

Supplement: Supplementary file 2 [file Image_2.TIF]

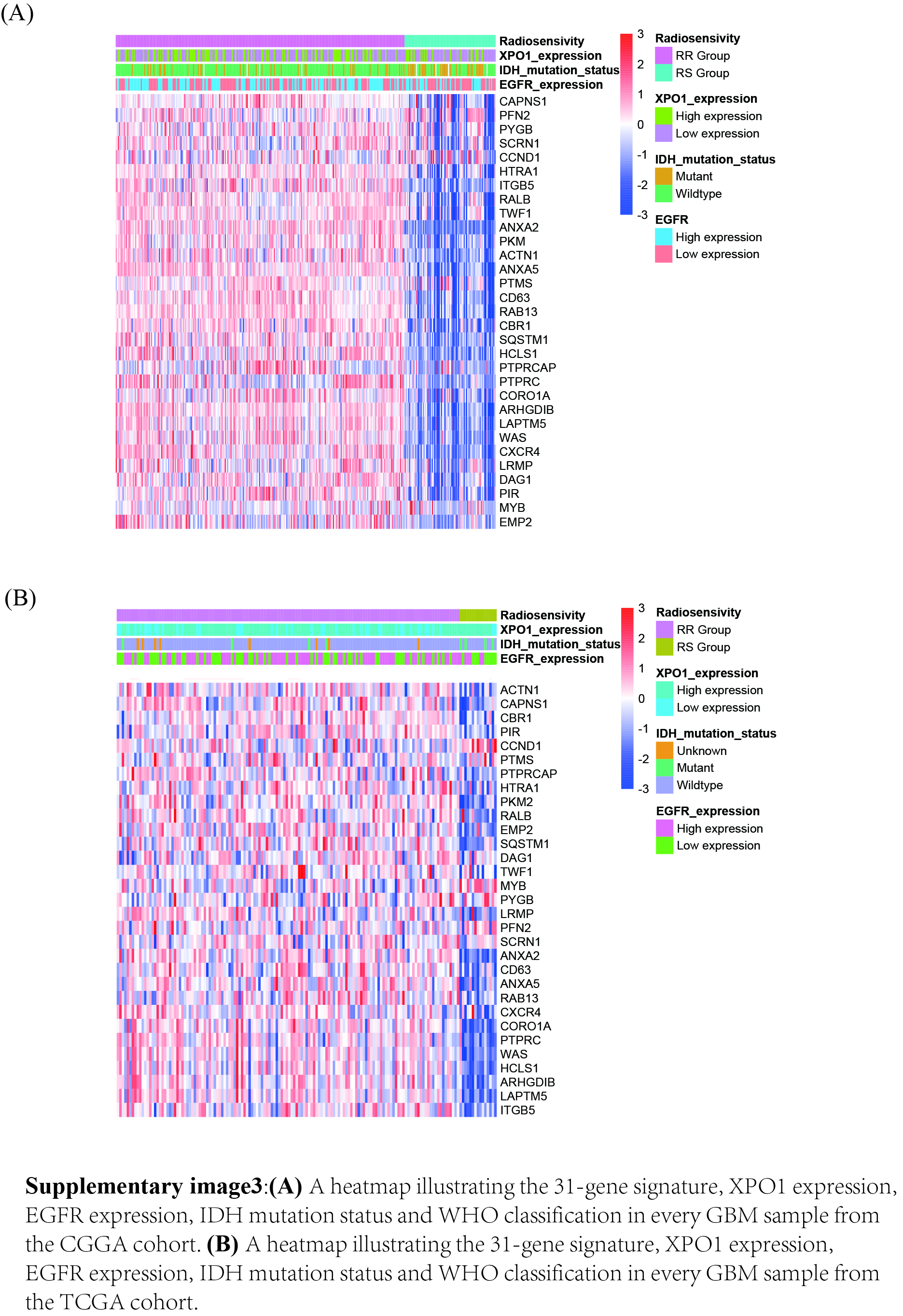

Supplement: Supplementary file 3 [file Image_3.TIF]
